# Retroperitoneal Schwannoma: A Rare Case

**DOI:** 10.1155/2011/465062

**Published:** 2011-07-14

**Authors:** Murat Kalaycı, Ümit Akyüz, Alp Demirağ, Bengi Gürses, Ferda Özkan, Özcan Gökçe

**Affiliations:** ^1^Department of General Surgery, Faculty of Medicine, Yeditepe University, Turkey; ^2^Division of Gastroenterology, Department of Internal Medicine, Faculty of Medicine, Yeditepe University, Turkey; ^3^Department of Radiology, Faculty of Medicine, Yeditepe University, Turkey; ^4^Department of Pathology, Faculty of Medicine, Yeditepe University, Turkey

## Abstract

*Introduction*. Schwannomas are quiet rare in the retroperitoneal region. Here, we describe an incidentally detected retroperitoneal schwannoma in the abdominal computerized tomography (CT) of a patient with acute appendicitis. *Case Presentation*. A 38-year-old woman was admitted to the emergency service with the complaints of progressive abdominal pain and nausea for the last 24 hours. Abdominal examination was compatible with acute abdomen. Acute appendicitis was diagnosed by CT. During CT evaluation, a round shaped soft-tissue mass at the retroperitoneal area inferior to the right kidney was detected, The mass was resected and histology revealed schwannoma. *Conclusion*. Rare tumoral lesions with benign course such as schwannoma can be detected incidentally.

## 1. Introduction

Primary tumors of the retroperitoneal region are quiet rare, and schwannomas comprise only 1–10% of them. Schwannomas originate from Schwann cells of the peripheral nerve fibers and are usually located in the head, neck, and flexor surfaces of the extremities. Schwannomas are quiet rare in the retroperitoneal region. Among all schwannomas, only 0.7% of benign ones and 1.7% of malignant ones are reported to be located in the retroperitoneal region. The majority of retroperitoneal schwannomas are benign in nature although malignant ones have also been reported [1–3]. 

Here, we describe an incidentally detected retroperitoneal schwannoma in the abdominal computerized tomography (CT) of a patient with acute appendicitis.

## 2. Case

A 38-year-old woman was admitted to the emergency service with the complaints of progressive abdominal pain and nausea for the last 24 hours. Physical examination revealed rebound abdominal tenderness at right lower quadrant. Laboratory tests showed increased white blood cell count (WBC) and mildly elevated erythrocyte sedimentation rate (ESR). Pelvic ultrasound (US) was not successful due to abundant gas. Therefore, CT (with a 64-slice scanner; Philips Brilliance 64, Best, NL after intravenous administration of nonionic contrast material) was performed to confirm acute appendicitis. 

**Figure 1:**
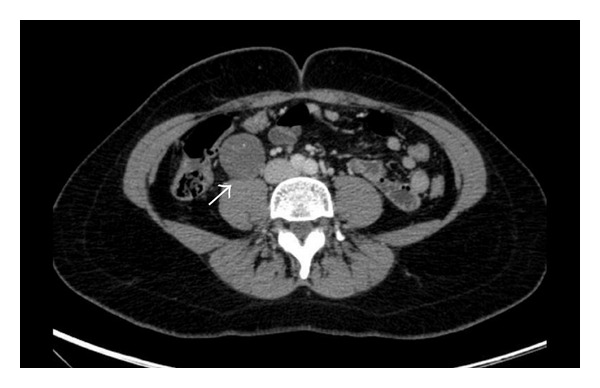
Axial CT image demonstrated an incidental retroperitoneal mass on the right side with minimal heterogenous contrast enhancement.

**Figure 2:**
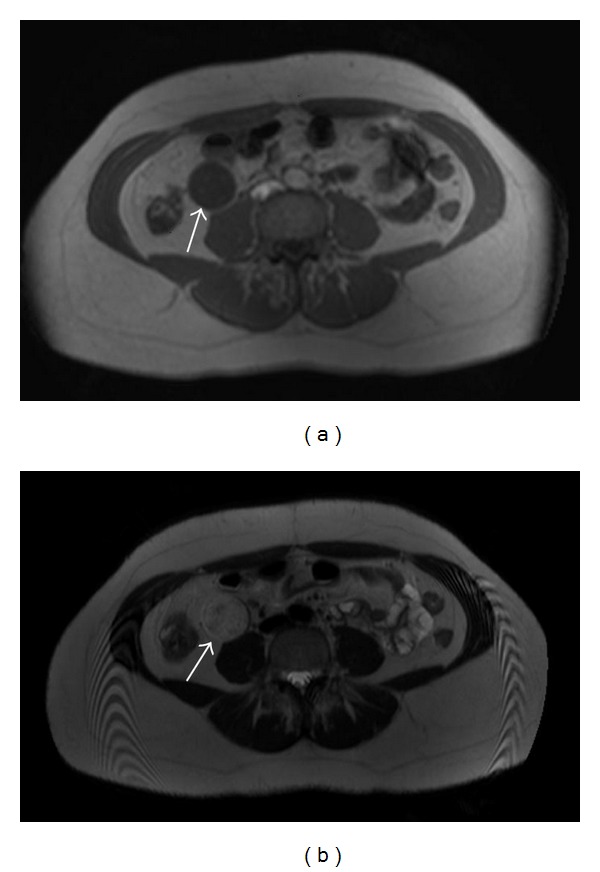
The retroperitoneal mass is hypointense on T1W (a) and heterogeneously hyperintense on T2W (b) MRI images.

CT revealed increased diameter of appendix (13 mm) with contrast enhancement and periappendicial fat stranding consistent with inflammation. The diagnosis of acute appendicitis was confirmed with CT. During CT evaluation, a round shaped soft-tissue mass 4.5 × 3.5 cm in diameter, with minimal heterogeneous contrast enhancement at the retroperitoneal area inferior to the right kidney, was detected (Figure 1). There were a few punctate calcifications inside the lesion on precontrast images. To further characterize the lesion, abdominal magnetic resonance imaging (MRI) was performed with a 3 Tesla scanner (Philips Intera Achieva, Best, NL) with Torso coil. The lesion was located just anterior to the iliopsoas muscle and inferior to the right kidney, was hypointense on T1 and heterogeneously hyperintense on T2 weighted images with moderate heterogenous contrast enhancement (Figures 2(a) and 2(b)). Owing to the location and signal characters, the presumptive diagnosis was a neurogenic or a fibrous tumor. Considering the possibility of a schwannoma, the presence of multiple associated schwannomas is eliminated with MRI examination of the whole spinal cord. 

**Figure 3:**
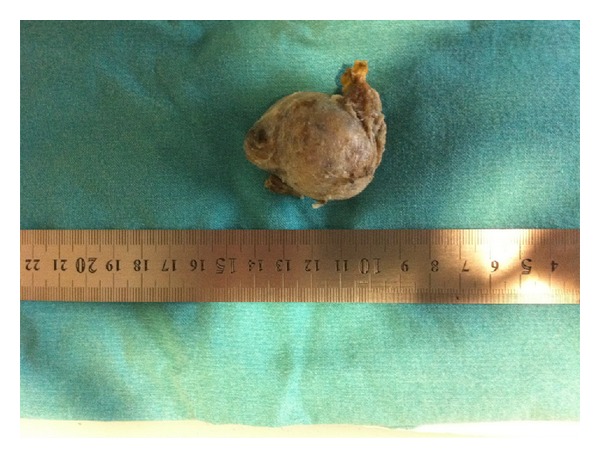
Resected 5 × 6 × 5 cm in diameters mass.

After midline abdominal incision, the abdomen was explored, and acute appendicitis was diagnosed. The right colon was mobilized and 5 × 6 × 5 cm in diameters mass was found. The mass was localized just above the right psoas muscle, lateral to the vena cava inferior and inferior to the right kidney, and also showed a close proximity to nerves ilioinguinalis and femorolateralis, and the mass was resected (Figure 3).

**Figure 4:**
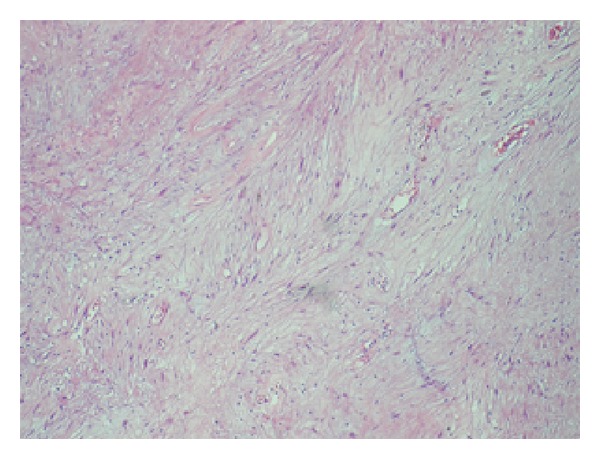
H&E, ×40; Antoni B areas characterized with less cellular, loosely textured Schwann cells.

Histopathologic examination of the lesion revealed a tumor composed of two different patterns characterized with cellular compact areas and loosely textured less cellular areas (Figure 4). Tumor cells were strongly and diffusely expressed S-100 protein, immunohistochemically. CD 117 (C-Kit), smooth muscle actin (SMA), and desmin were negative.

## 3. Discussion

Schwannomas are nerve sheath tumors that are mostly benign in nature. These neoplasms are usually seen in adult population between the ages of 20 and 50. Symptomatology of benign schwannomas is highly nonspecific and depends on the location and size of the lesion. 

Retroperitoneal region is a rare location for schwannomas except in patients having Von Recklinghausen's disease. It is also noteworthy to mention that malignant degeneration particularly takes place in association with Von Recklinghausen's disease. In general, since the retroperitoneal space is rather large and flexible, the diagnosis of retroperitoneal schwannomas is often delayed, and the lesion reaches a significant size at the time of diagnosis. The most common symptoms are abdominal pain and distention. Depending on the location of the lesions, a variety of symptoms such as secondary hypertension, hematuria, and renal colic have also been reported. 

Schwannomas are located typically eccentric in relation to the nerve of origin. This finding could clearly be seen both macroscopically and microscopically in our patient. They often have a true capsule which is composed of epineurium. In general, schwannomas are seen as hypointense on T1 and hyperintense on T2 weighted MR images. Calcification has been reported to have an incidence of only 23% in retroperitoneal schwannomas, which has been observed in our patient on precontrast CT images. Cystic degeneration has been reported more frequently in retroperitoneal schwannomas with an incidence of up to 66%. 

In general, MRI is regarded as the diagnostic modality of choice in the evaluation of retroperitoneal tumors. MRI allows better evaluation of the origin, extent, and internal composition of these lesions. There are a few well-known imaging characters for schwannomas which are mainly target sign and fascicular sign. However, these typical signs are not seen frequently in retroperitoneal schwannomas. The “fascicular sign” stands for the the appearance of bundles which is a general property of neurogenic tumors. On the other hand, the “target sign” is the presence of hypointense center and hyperintense periphery on T2 weighted MRI. The lesion presented in this paper does not exert any of the above-mentioned typical diagnostic signs [4, 5]. Hence, we believe that for the preoperative diagnosis of retroperitoneal schwannomas, a high index of suspicion is mandatory, especially in the absence of characteristic imaging features, as in our patient. 

The differential diagnosis for retroperitoneal schwannomas includes other neurogenic tumors such as paraganglioma and pheochromacytoma as well as, liposarcoma and malignant fibrous histiocytoma. In addition to those, if the retroperitoneal schwannoma contains considerable amount of cystic degeneration, retroperitoneal cystic masses such as hematoma and lymphangioma should also be included in the diagnostic checklist.

Although rare, malignant counterparts of schwannomas also exist. Detection of a malignant schwannoma is highly important, since it will affect the treatment strategy. From the radiologist's point of view, malignant schwannomas have irregular contour and tend to show invasion to the adjacent structures. The retroperitoneal lesion in our patient had regular borders without any sign of adjacent organ invasion, which were highly suggestive of a benign lesion radiologically.

For the surgical treatment of retroperitoneal schwannomas, the current approach is endoscopic-assisted minilaparotomy. Aggressive surgery is not indicated for benign retroperitoneal schwannomas. Although local resection is generally enough, metastatic cases have been reported after resection [1]. Therefore, followup is important for this patient.

## 4. Conclusion

We presented a rare type of retroperitoneal tumor which was detected incidentally in a patient diagnosed with acute appendicitis. Rare tumoral lesions with benign course such as schwannoma can be detected incidentally.
